# Deep arsenic mobilization in karst soils: effects of pedogenesis and grazing

**DOI:** 10.7717/peerj.21371

**Published:** 2026-06-08

**Authors:** Ruiyin Han, Qian Zhang, Xing Gao

**Affiliations:** 1 State Key Laboratory of Lithospheric and Environmental Coevolution, Institute of Geology and Geophysics, Chinese Academy of Sciences, Beijing, China; 2 University of Chinese Academy of Sciences, Beijing, China; 3 Institute of Geographic Sciences and Natural Resources Research, Chinese Academy of Sciences, Beijing, China

**Keywords:** Arsenic dynamics, Land utilizations, Risk assessment, Karst area, Southwest China

## Abstract

Arsenic (As) contamination poses substantial ecological and human health concerns in karst regions due to high geochemical background levels and enhanced mobility associated with carbonate weathering and thin soil cover. To investigate the vertical distribution, sources, and environmental risks of soil As under different anthropogenic land uses, we analyzed soil profiles from three representative ecosystems—secondary forest, abandoned cropland, and grazing shrubland—in a typical karst area of Southwest China. The As contents were identified between secondary forest (19.00 ∼ 41.17 mg/kg), abandoned cropland (22.55 ∼ 34.47 mg/kg), and shrubland (14.19 ∼ 34.46 mg/kg), with no significant differences, indicating a predominantly geogenic origin from carbonate bedrock weathering. Long-term cultivation homogenized surface soil As through plowing and crop uptake, whereas grazing introduced additional organic-bound As *via* livestock excreta, promoting downward migration and accumulation in deeper soil layers. Ecological risk assessment, using contamination and enrichment indices, revealed low to moderate potential risks (contamination factor: 0.71 ∼ 2.06; enrichment factor: 0.76 ∼ 2.34). Health risk assessment indicated that non-carcinogenic risks (hazard index, HI: 0.09 ∼ 0.21) were within acceptable limits for all exposure pathways, while carcinogenic risks for children (carcinogenic risk (CR): 0.11 ∼ 0.21) approached the threshold of concern. Despite overall low risk levels, the high mobility of As and its potential co-mobilization with other toxic elements underscore the need for continued monitoring. These findings highlight the importance of sustainable land-use practices to mitigate As mobilization and safeguard soil quality in vulnerable karst ecosystems.

## Introduction

As a fundamental environmental media, soil is an important natural resource for the development of any nation which serves as the primary reservoir of nutrients and potential contaminants influencing environmental health. The quality of soil for production depends on its sustainable supply of plant nutrients (Fartyal et al., 2025b). Soil gives support in terms of moisture, nutrient and anchorage to plants to grow effectively on the one hand, and on the other, plants provide protective cover for soil, suppresses soil erosion as well as helps to maintain soil nutrient through litter accumulation and subsequent decay (Awasthi et al., 2022; Gonzalez-Quiñones et al., 2011; Pandey et al., 2024). The land use systems effectively influence fertility and stability of an ecosystem and has been accepted widely as a vital source of nutrients due to its quick turnover (Bargali, Padalia & Bargali, 2019; Fartyal et al., 2025a; Padalia et al., 2018). Binary hydrological structure (rapid conduit flow/slow matrix flow) combined with thin soil layers (< 2 m), exhibit limited contaminant retention capacity, rendering groundwater in karst ecosystems highly vulnerable to contamination by potentially toxic elements (PTEs, *e.g.*, Zn, Cr, Cd, Fe, Hg, and As) contamination in the karst ecosystem (Chu et al., 2025; Jia et al., 2020). Noteworthy, the adverse effects of agricultural activities on soil can persist over long periods and are difficult to reverse through natural recovery processes by itself (Han, Li & Tang, 2015; Han & Liu, 2004; Liu, Han & Zhang, 2020; Zhang et al., 2023). In Southwest China’s karst trough valleys, cationic heavy metals (*e.g.*, Zn^2+^, Cr^3+^) are predominantly retained within shallow soil horizons (< 80 cm), governed primarily by adsorption onto clay minerals and co-precipitation with Fe/Mn oxides (Han, Zhang & Xu, 2023b; Zhang et al., 2021). This cationic retention paradigm have been evidenced by: (1) enrichment of Zn contents in topsoils (difference > 40 mg/kg in the vertical profile) relative to subsoil, indicating Zn adsorption in surface horizons (Han, Liu & Xu, 2024a), (2) Hg-Fe co-variation (*R* = 0.88) in surface soils (Qu et al., 2021). This accumulation pattern leads a large proportion of metal elements remain in soil (Cr, Cd, Pb distribution (I_geo_ < 0)) without deep enrichment (Han & Xu, 2022). In contrast, As-predominantly occurring as mobile anionic species (AsO_4_^3−^/AsO_3_^3−^): exhibits divergent geochemical responses to anthropogenic disturbance (Bowell, 1994; De Mello et al., 2007). Soil As is mainly derived from the weathering of As-bearing bedrock, and its mobility is further enhanced by high hydraulic conductivity and well-developed preferential flow pathways in the karst area (Jia et al., 2020; Li et al., 2022). This enhances contamination risks to groundwater-dependent ecosystems (Zhang et al., 2017).

Although the As content is usually not the highest among PTEs, it frequently dominates ecological risk due to its higher toxicity (Lai et al., 2024; Zhang et al., 2025). Rapid urbanization and agriculturalization can trigger As accumulation in cultivated soils through the application of fertilizers and pesticides. Excessive As levels in soil not only disrupt soil microbial communities, but also change soil organisms and further adverse to soil structure and fertility. These intensive agricultural activities will lead an obvious increase of As on regional scale. In fact, excessive soil As will directly cause soil productivity and crop quality reduction and further threaten the human food supply (Gong et al., 2020). As is a non-essential element for biological growth, which disrupts critical biological processes (*e.g.*, chlorophyll synthesis in plants), while posing serious risks to human health (*e.g.*, diabetes, cancer, cardiovascular and neurological diseases) (Chu et al., 2025; Upadhyay et al., 2019; Zhang et al., 2025). Additionally, As can cause economic losses to the livestock industry through the grass-cattle system, while leading to secondary exposure to humans through the livestock products (Valani et al., 2021).

The content and influence factors of soil As are important to explore the biotoxicity of As on a regional scale. Assessing soil As enrichment levels and exploring the As dynamics can optimize environmental management and improve soil quality. Agricultural activities (*e.g.*, grazing) generally accompany with redox dynamics *via* organic matter accumulation or Fe reduction (Han, Zhang & Xu, 2023b; Liu, Han & Zhang, 2020), which further control soil As transport and transformation (Malakar et al., 2021). Previous studies basically focused on the pollution of soil under As mining or industrial emissions because worker may be exposed to As dust through skin contact or inhalation. (Chowdhury et al., 2018). However, As from agricultural soil can threaten human health not only through direct contact, but also through dietary intake because of the strong enrichment ability of crops (Gong et al., 2020; Li et al., 2022). The responses of soil As dynamics on agricultural activities should pay more attention. In addition, because of the regional environmental effects caused by the possible diffusion of As, the monitoring and assessment of As in abandoned agricultural land should not be ignored. To reveal the behavior mechanism of As in agricultural land and abandoned agricultural land can provide scientific basis for optimizing regional environmental management. However, critical uncertainties persist: (1) whether grazing *vs.* geogenic weathering plays the dominant role in As redistribution; (2) whether pore-scale constraints lead to anion-selective transport beyond cationic retention; and (3) the likelihood of groundwater potential contamination resulting from soil As vertical migration under the influence of human activities. To resolve these knowledge gaps, this study aims to (1) explore the distribution and enrichment patterns of As in typical karst soils under different land uses, (2) decipher the mechanisms of anion-specific transport and transformation under different land-uses, and (3) assess ecological contamination risk and provide valuable basis for optimizing land management and environmental protection in the karst area.

## Materials & Methods

### Study area and sampling strategy

**Figure 1:**
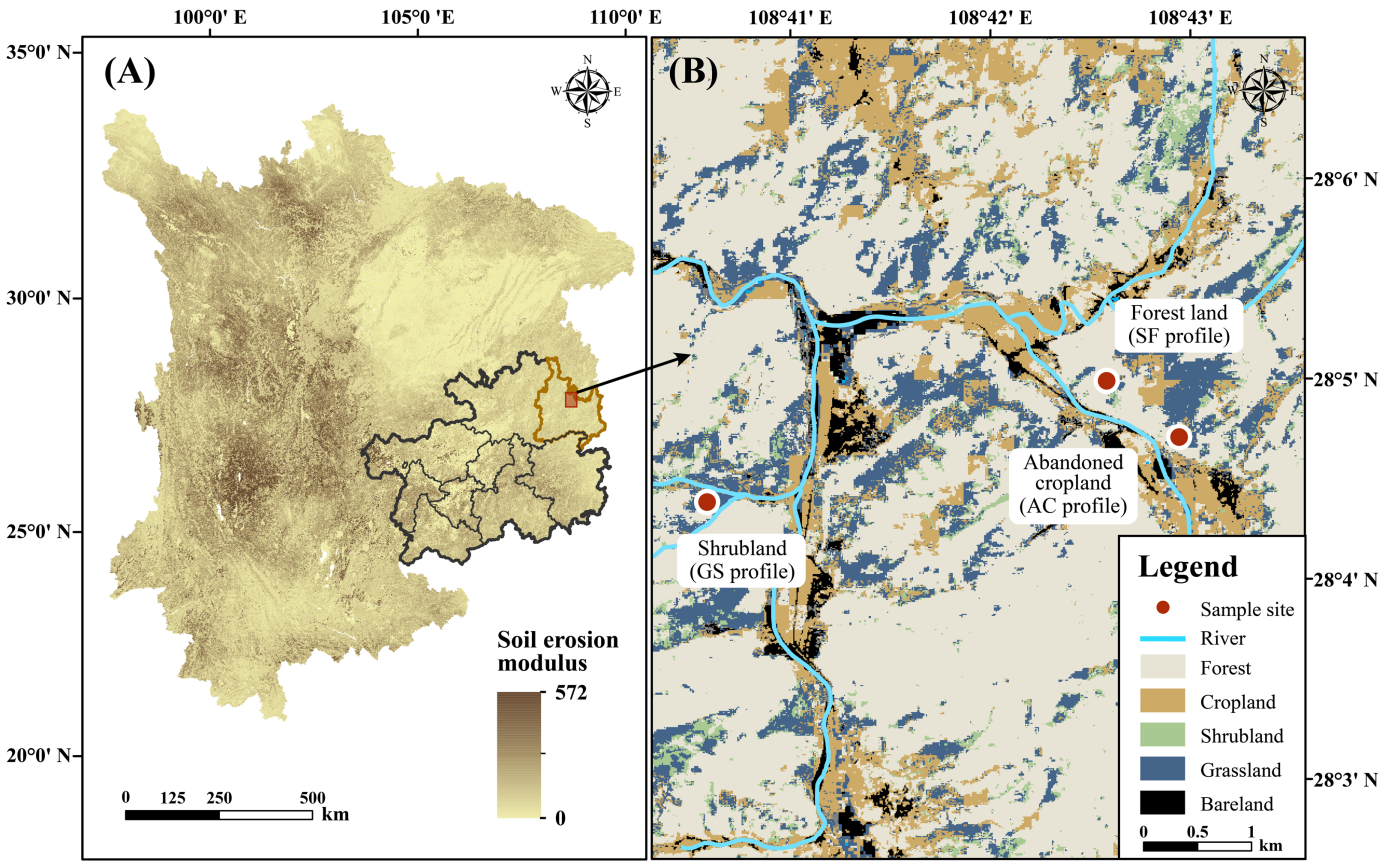
Sampling sites in the study area, (A) Soil erosion model in the southwestern China in 2015 (Wang, Zhu & Chen, 2021a; Wang, Zhu & Chen, 2021b), (B) land uses in the study area.

The sampling sites are in Yinjiang County (27°35′∼28°21′N, 108°18′∼108°48′E, Fig. 1, Table S1), a representative karst trough valley in Guizhou Province, Southwest China. This region exhibits typical karst geomorphology with high hydraulic connectivity, thin soil cover (< 2 m), and significant elevation gradients of > 500 m (Han et al., 2020; Qu & Han, 2022). The largest proportion of covered land in the total area is forest along with abundant mountains. Followed by the farmland, which occupied more than 18,000 hectares with an agricultural output of more than 144,000 tons in Yinjiang County in 2016 (Statistics, 2017). Three key land-use types were examined: secondary forest (SF): minimally disturbed natural ecosystem; abandoned cropland (AC): 50-year cultivation history, abandoned for 3 years; grazed shrubland (GS): active goat grazing for more than 5 years. Soil profiles (SF: *n* = 20; AC: *n* = 16; GS: *n* = 11) were sampled in September 2016 following protocols detailed in Han & Xu (2022). At each land-use type, independent sampling sites were selected, and three duplicate soil profiles were excavated at each site to characterize within-site variability. Because soil properties in karst areas show strong spatial heterogeneity, especially along the vertical direction, the distance between the three profiles at the same site was kept within 1 m to ensure that they represented the same pedogenic environment rather than independent sites. Soil samples were collected at 5 cm intervals above 20 cm depth and at 10 cm intervals below 20 cm depth. Samples from the three duplicate profiles at the same depth were composited to form one representative sample for that depth at each site. Bedrock samples (*n* = 2) from parent limestone were concurrently obtained.

### Integrated datasets and new As analyses

This study synthesized some published geochemical data: (1) Fe/Zn isotopic compositions (*δ*^56^Fe, *δ*^66^Zn) and associated elemental concentrations (Fe, Zn, Cr, Hg) (Han, Liu & Xu, 2024a; Han & Xu, 2022; Han, Zhang & Xu, 2023b; Qu et al., 2021), and (2) soil physicochemical properties (pH, soil organic carbon (SOC), texture) (Han, Zhang & Xu, 2023a; Han, Zhang & Xu, 2024b). Total As contents were determined by monochromatic energy-dispersive X-ray fluorescence (MED-XRF, E-max500, JP Scientific, China). A previous study has demonstrated that the testing of inorganic elements in soils by MED-XRF was better than transitional ED-XRF and wavelength dispersive X-ray fluorescence spectrometer (WD-XRF) (Xing et al., 2022). Quality control and quality assurance were performed by validating detection limits (0.5 mg/kg) and precision (± 5%) with the procedural blank and standard reference material (GBW07447 and GBW07449). All measurements were completed at the Institute of Geographic Sciences and Natural Resources Research, Chinese Academy of Sciences.

### Data treatment

#### Pedogenic As migration

PTEs easily migrate vertically with leaching by precipitation or agricultural irrigation. This transport characteristics are assessed by the index of relative topsoil enhancement (RTE, As contents in topsoil over that in subsoil) (Li et al., 2022; Tsadilas & Shaheen, 2010). The structure and characteristics of bedrock will remain during the weathering to follow the mass balance in a profile without anthropogenic disturbance. To clarify the geochemical characteristics of As during the carbonate rock weathering in the karst region, the index of mass balance (ΔT) is applied to evaluate the degree of weathering and leaching. The ΔT values are calculated to refer to a representative element such as Ti, Zr, and Th (Brimhall et al., 1988). In consideration of the lower contents of Zr in rocks and the easier soluble in water of Ti (Nesbitt & Wilson, 1992), the Th was chosen to calculate ΔT with limited variations in the study area.

\begin{eqnarray*}\mathrm{RTE}={\mathrm{M}}_{\mathrm{i}}/{\mathrm{M}}_{\mathrm{i}-1} \end{eqnarray*}

\begin{eqnarray*}\Delta \mathrm{T}= \left[ \left( {\mathrm{As}}_{\mathrm{s}}\times {\mathrm{Th}}_{\mathrm{r}} \right) / \left( {\mathrm{As}}_{\mathrm{r}}\times {\mathrm{Th}}_{\mathrm{s}} \right) -1 \right] \times 100\%, \end{eqnarray*}

where the RTE represents the leaching ratios of As in the *i* soil layer; and M represents the measured As contents in the corresponding soil layer. The RTE value is higher than 1 indicating the mobility rate of soil As is low and accumulates in soil, vice versa. The *s* and *r* represent the corresponding element contents of soils and rocks. The value of ΔT > 0 indicates the enrichment of As in soils relative to that in bedrock, while ΔT < 0 indicates the defective of As in soils relative to that in bedrock, and ΔT = 0 indicates neither enrichment nor defective of As in soils relative to that in soils.

#### Ecological risk assessment

Ignoring soil As risks may lead to irreversible groundwater degradation. Although the As in soils is generally considered a microelement, the toxicity is non-negligible and the toxic-response factor of As reaches 10 (Hakanson, 1980). Ecological risk assessment of soil As is critical for safeguarding groundwater resources due to its high mobility and persistent toxicity. As can migrate through soil profiles *via* leaching and hydrological pathways, potentially contaminating aquifers. The formula of contaminated factor and potential ecological risk of the interest element are as follows:

\begin{eqnarray*}{\mathrm{C}}_{\mathrm{c}}={\mathrm{C}}_{\mathrm{s}}/{\mathrm{C}}_{\mathrm{r}} \end{eqnarray*}

\begin{eqnarray*}{\mathrm{E}}_{\mathrm{As}}=10\times {\mathrm{C}}_{\mathrm{c}}, \end{eqnarray*}

where the C_c_ means the contaminated factor of As, the C_s_ and C_r_ mean the measured content of As and the reference value of As in soils of Guizhou Province, respectively. The E_As_ represents the potential ecological risk of As. The value of C_c_ ≤ 0.7 indicates a great ecological environment, 0.7 < C_c_ ≤ 1.0 indicates a safe ecological environment, while 1.0 < C_c_ ≤ 2.0 indicates slight contamination (Gujre, Rangan & Mitra, 2021). A value of E_As_ < 40 indicates a slight ecological risk, while an E_As_ value higher than 40 indicates a noteworthy of ecological risk (Gujre, Rangan & Mitra, 2021).

#### Health risk assessment

Humans can be exposed to soil As through three primary pathways: ingestion, inhalation, and direct skin contact (Adeniji, Okoh & Okoh, 2019; Alharbi et al., 2023). The diverse exposure routes underscore the necessity for a comprehensive health risk assessment. An empirical model was utilized to evaluate the non-carcinogenic health risks (USEPA, 2011):

\begin{eqnarray*}{\mathrm{CDI}}_{\mathrm{dermal}}= \left( \mathrm{C}\times \mathrm{SA}\times \mathrm{EF}\times \mathrm{ED}\times \mathrm{AF}\times \mathrm{ABS}\times 1{0}^{-6} \right) / \left( \mathrm{BW}\times \mathrm{AT} \right) \end{eqnarray*}

\begin{eqnarray*}{\mathrm{CDI}}_{\mathrm{ing}}= \left( \mathrm{C}\times \mathrm{IngR}\times \mathrm{EF}\times \mathrm{ED}\times 1{0}^{-6} \right) / \left( \mathrm{BW}\times \mathrm{AT} \right) \end{eqnarray*}

\begin{eqnarray*}{\mathrm{CDI}}_{\mathrm{inh}}= \left( \mathrm{C}\times \mathrm{InhR}\times \mathrm{EF}\times \mathrm{ED} \right) / \left( \mathrm{BW}\times \mathrm{AT}\times \mathrm{PEF} \right) \end{eqnarray*}

\begin{eqnarray*}\mathrm{HQ}=\mathrm{CDI}/\mathrm{RfD}, \end{eqnarray*}

where the chronic daily intake (CDI) serves to quantify the daily health impacts on the population resulting from dermal, ingestion, and inhalation exposure to As in soils, and hazard quotient (HQ) uses to estimate non-carcinogenic health risks posed by As in soils. If the value of HQ lower than 1 represent no non-carcinogenic risk threaten human health, otherwise indicated the occurrence of non-carcinogenic risk for exposed residents. C represents As contents in soil; SA represents exposed skin surface; EF represents exposure frequency of people; ED represents exposure duration; AF represents adhesion coefficient of skin; ABS represents absorption factors of skin; BW represents body weight; AT represent average time; IngR and InhR represent ingestion and inhalation rates of soil, respectively; PEF represents particulate emission factor; RfD represents reference dose.

Additionally, risk assessment of As needs to consider carcinogenic risk (CR), which indicates the probability of cancer development during people lifetime through soil As exposure (USEPA, 2011):

\begin{eqnarray*}\mathrm{CR}=\mathrm{CDI}\times \mathrm{CSF}, \end{eqnarray*}

where CSF represents carcinogenic slope. The value of CR lower than 10^−6^ indicates inconspicuous cancer risk on health, the value between 10^−6^ and 10^−4^ indicates moderate risk, while the value higher than 10^−4^ indicates considerable higher cancer risk (Adeniji, Okoh & Okoh, 2019).

All parameters were referenced by, while part of them (such as BW, ABS, SA) were corrected with Chinese official reports (Ministry of Ecology and Environment P, 2013; Ministry of Ecology and Environment P, 2016). Detail values of each parameter were listed in Table S2. All measured data were analyzed by SPSS 25.0 (IBM SPSS Statistics, Chicago, IL, US), and graphed by Origin 2021 (OriginLab, Northampton, MA, USA). The map data were processed by ArcMap 10.8 (Esri, Redlands, CA, USA).

## Results

**Figure 2:**
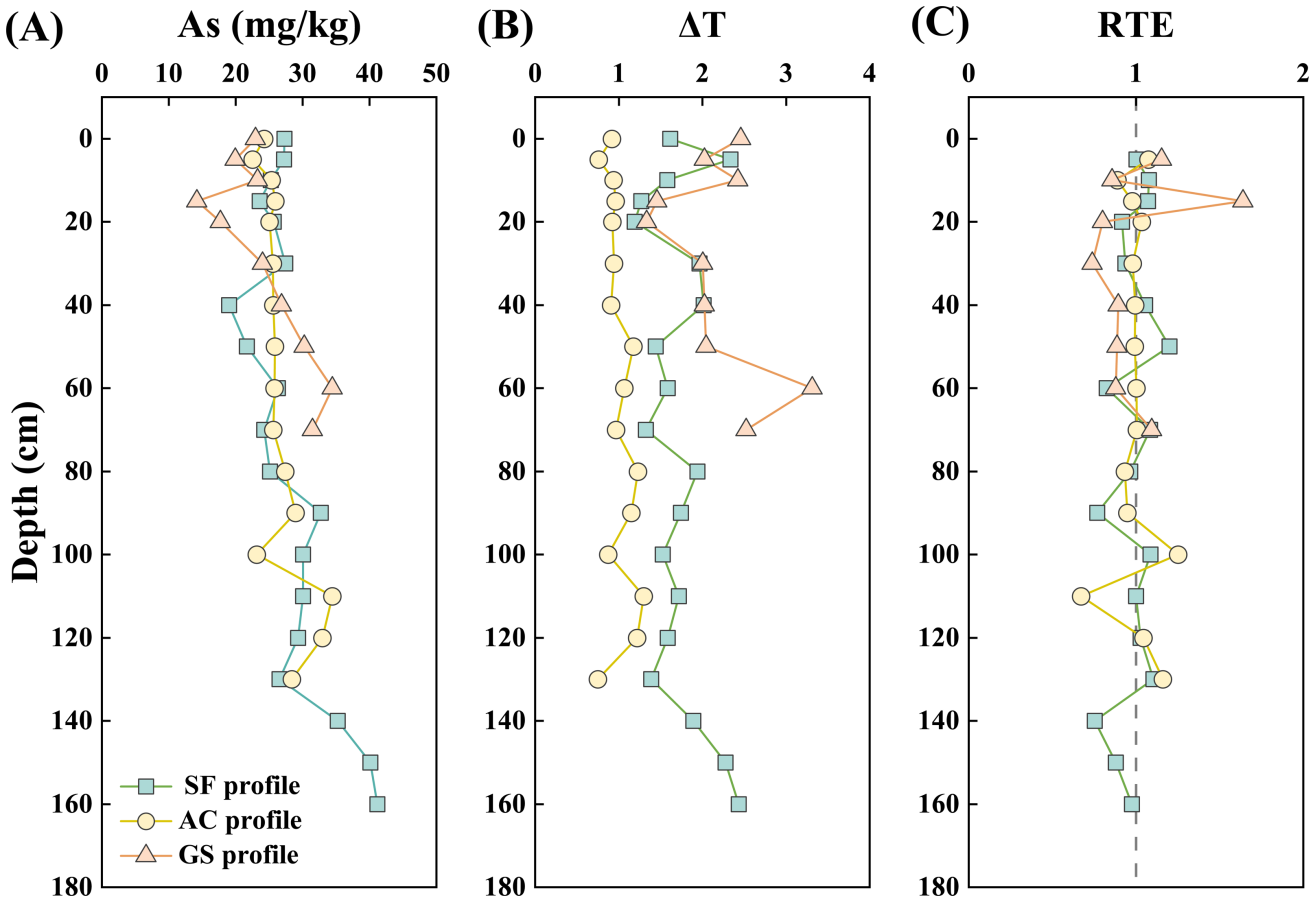
Vertical distributions of As contents (A), ΔT values (B), and RTE values (C) in the study area.

Results showed that all soil profiles were dominated by silt fraction (> 70%) with moderate to extreme weathering. The slightly alkaline soil environment was observed in the SF profile, while the slightly acidic soil environment was found in the AC profile, and the relative medium soil environment was found in the GS profile. The As contents and related parameters are listed in Table S3. The detail information have been reported by Han, Liu & Xu (2024a). The vertical variations of As in the three profiles were presented in Fig. 2. The average level of As contents in the study area was higher than that in the background of Guizhou Province (20.00 mg/kg), but lower than the average level of As in the agricultural soil of Guiyang city (29.11 mg/kg) (CNEM Centre, 1990; Li et al., 2022). The As contents in three profiles decreased in the sequence: SF profile (30.09 ± 11.09 mg/kg) > AC profile (28.51 ± 5.96 mg/kg) > GS profile (24.33 ± 10.14 mg/kg). The As contents in the AC profile experienced long-term cultivation were higher than that in the cropland soils in China (13.90 ± 6.60 mg/kg) (CNEM Centre, 1990). Along the profile, the highest value of As was observed in the deeper soils of the SF (160 cm), AC (110 cm), and GS (60 cm) profiles. The As contents limited varied in the upper soil and showed an increasing trend below 40 cm in the SF profile, while the As contents showed an increasing trend from surface to deeper soil in the GS profile; and the As contents showed narrow variation with soil depth increasing in the AC profile, indicating possible redistribution influenced by grazing activities. There were no obvious changes in As contents in the topsoil (0 to 15 cm) in the GS profiles, while there was a noticeable increase below 20 cm. This pattern suggests significant accumulation of As in deeper layers, contrary to the typical surface enrichment observed for many cationic heavy metals (*e.g.*, Cr, Cd, Pb) in similar karst environments (Han & Xu, 2022).

## Discussion

### Anomalous depth enrichment of As during Pedogenesis

**Figure 3:**
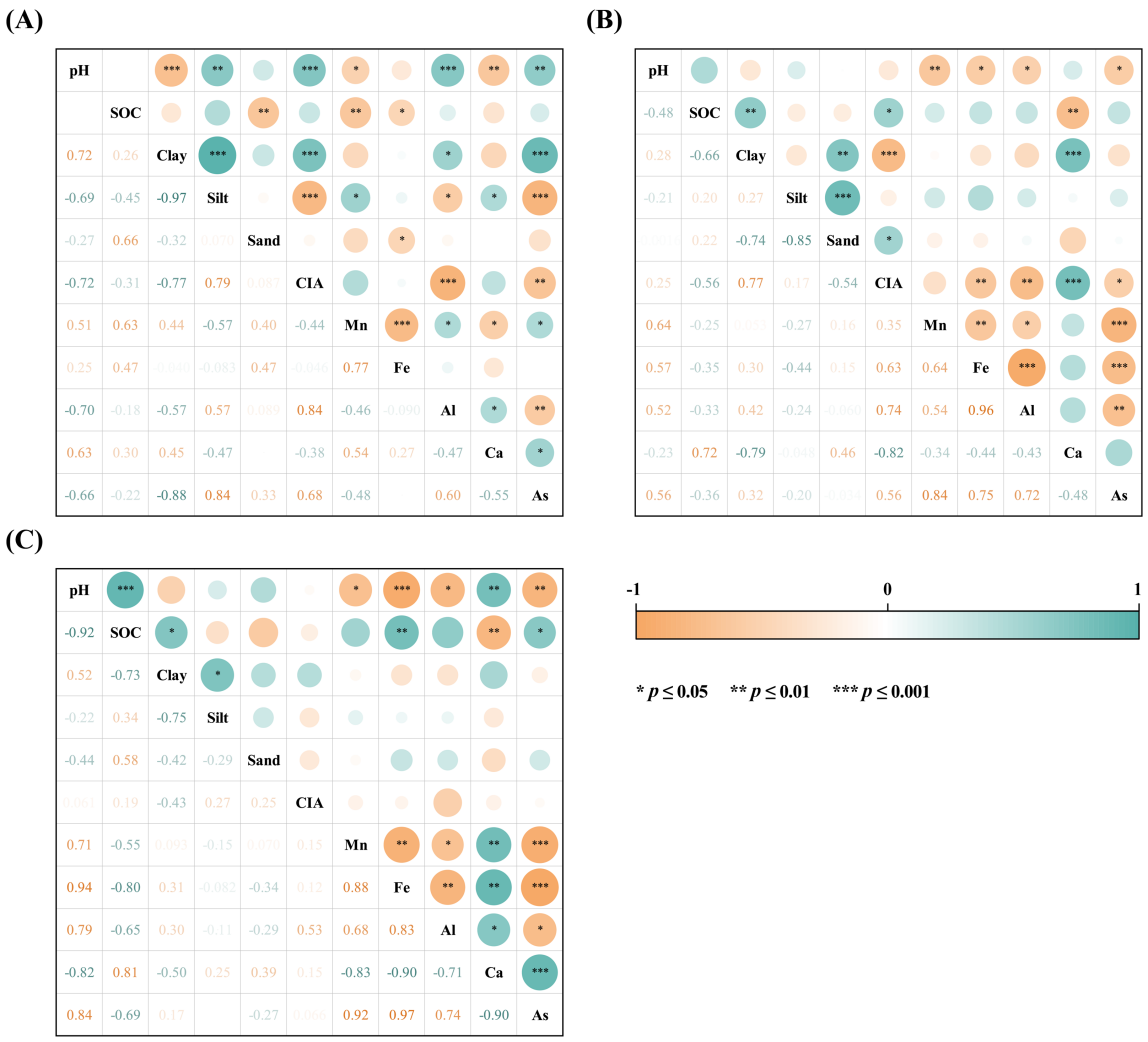
Correlation analysis among As contents and related soil parameters in the SF (A), AC (B), and GS (C) profiles.

Clear cut variations in the soil As have been marked along the sites and soil depth. These variations may be linked to several environmental factors. In any land use systems soil aggregates vary in space and time because of variation in vegetation cover, litter type, topography, climate, weathering processes, and microbial activities (Manral et al., 2022; Paudel & Sah, 2003) and several other biotic and abiotic factors (Bisht et al., 2025; Pandey et al., 2024). In the highly dissected landscapes, bioclimatic conditions change rapidly and may vary within short distances resulting in a pronounced heterogeneity of soil aggregates and their chemical and physical properties (Bargali, Padalia & Bargali, 2019; Bäumler, 2015). Variations in plant functional traits and litter type may also help to change in diverse abiotic and biotic environments (Naidu et al., 2026; Silva et al., 2019), affect and alter ecosystem processes (Crutsinger et al., 2006; Manral et al., 2022). The natural rock weathering possibly becomes the dominating contributor of soil As in the study area. The As accumulation may be related to the high geological background in Guizhou Province (As level: 20 mg/kg) (CNEM Centre, 1990), while the widespread distribution of carbonate rocks also improves As accumulation in the study area. The positive correlations between chemical index of alteration (CIA) values and As contents were only found in SF and AC profiles (Fig. 3), indicating the primary contribution of bedrock on As. While the complex contributors and pedogenic processes may act on the soil As GS profile. The depth-enrichment pattern of As contrasts sharply with the retention behavior of cationic metals such as Zn and Fe previously reported in the same catchment (Han, Liu & Xu, 2024a; Han, Zhang & Xu, 2023b). Zn and Fe typically show surface enrichment due to organic matter complexation, adsorption on Fe/Mn oxides, and biological cycling (Jaradat et al., 2009). In contrast, As often present as oxyanions (*e.g.*, AsO_4_^3−^), which exhibits higher mobility under certain pH and redox conditions, leading to leaching and deeper accumulation (De Mello et al., 2007). This divergence can be attributed to differences in geochemical affinity and mobilization mechanisms. Cationic metals are preferentially adsorbed onto negatively charged clay minerals and organic matter in surface horizons, arsenate anions are less retained in acidic to neutral soils and may be transported downward with percolating water (Masscheleyn, Delaune & Patrick Jr, 1991). Moreover, reducing conditions that may occur in deeper soil layers can promote the transformation of As^5+^ to the more mobile As^3+^. The adsorption of soil particles possibly not be the main controlling factor of As dynamics in the study area.

All values of ΔT were higher than 0, indicating that the soil As was richer than that of bedrock in the study area (Fig. 2). The values of RTE ranged narrowly in each profile (SL: 0.975 ± 0.225, AC: 0.960 ± 0.290, GS: 0.945 ± 0.205), excluding the soil at 15 cm layer (RTE = 1.64) of the GS profile. The relative stable variation of RTE values in the three profiles indicated the influence of original rock weathering on soil As vertical transportation is weak. Fe, Al, and Mn can be substituted for As in rocks by isomorphism, and As will precipitate with Fe, Al, and Ca in insoluble form during the weathering process reported by previous studies (Zhang et al., 2017). Figure 3 showed a significant positive correlation between As and Ca (*p* ≤ 0.001), and the enrichment of As in the 15 cm soil layer of the GS profile is closely related to higher contents of Ca (48.76 mg/kg, the highest value in the GS profile). Therefore, As compounds can be complex with Ca^2+^, promoting its retention of As in soil through co-precipitation with carbonates. The As contents showed positive correlations with Fe and Mn contents (*p* ≤ 0.001), and also positive correlations with soil pH (*p* ≤ 0.05) in the AC and GS profiles (Fig. 3). The distribution of As is strongly controlled by the co-precipitation complexes with metallic oxide, carbonates, or hydroxides under acidic conditions, and tends to lead by Fe-oxides adsorption as soil pH increases (Cancès et al., 2005; Sparks, Singh & Siebecker, 2024). A larger specific surface area and positive charge distribution in the surface of amorphous Fe-oxides lead stronger adsorption capacity of As (Ma, Cai & Tu, 2020). Even the proportion of amorphous Fe-oxides occupied less than 15% of the total soil Fe, which can absorb more than 80% As of the total soil As, highlighting their disproportionate role in As immobilization (Zhang et al., 2021). Furthermore, Mn can act as oxidizing agents, facilitating the transformation of As^3+^ to As^5+^ andFe^2+^ to Fe-oxides, thereby improving the adsorption and immobilization of As by Fe-oxides (Yang, Han & Jiang, 2021). However, grazing activities cause the soil environment to become more acidic, which hinders the precipitation of As.

### Grazing mediates reductive As mobilization

The SF profile with minimal anthropogenic disturbance, exhibits a clear and consistent increase in As concentration with depth, suggesting that natural pedogenic processes dominate As cycling in this soil. In contrast, the AC profile subjected to historical cultivation, shows a more uniform distribution in the plow layer (0 ∼100 cm), likely due to historical fertilization and tillage homogenizing the topsoil. The abrupt increase below 100 cm may indicate leaching of As from the surface over time. The GS profile, influenced by grazing, exhibits the highest variability, possibly due to bioturbation, manure deposition, and altered soil structure. The very low As value at 15 cm may reflect temporary immobilization *via* organic complexation or surface runoff, while the peak at 60 cm suggests accumulation in a less permeable layer or redox interface. Due to the strong mobility, As easily migrates downward during leaching and accumulates in deeper layer (Chowdhury et al., 2018). Exogenous inputs (*e.g.*, pesticides and insecticides) generally affect As concentrations mainly in the topsoil (0 ∼20 cm), with limited influence below ∼60 cm depth (Wang et al., 2016). As described previously, the As inputs from sustainable fertilizer application are generally lower than that of atmospheric deposition into the soils, and long-term accumulation of As-fertilizer is limited (Wang et al., 2016). In addition to competitive adsorption between phosphate and arsenates, more As is imported in the form of soluble fertilizer application and further absorbed by crops (Lee, Chiang & Houng, 1996; Upadhyay et al., 2019). Continuous plant uptake during crop growth may thus prevent significant As accumulation in the tilled layer, even when phosphate fertilizers containing trace As are repeatedly applied (Wang et al., 2016). Therefore, the higher level of As contents in the surface soil of AC profile experienced long-term cultivation have not been found. However, the As contents in the three soil profiles were close to the critical safe levels of As (< 30 mg/kg) for crops by soil quality criteria in China (Zhou, Teng & Liu, 2017). The variation of As contents in deep soil (below 60 cm soil layer) of the AC profile was similar to that in the SF profile, which were close in distance. Therefore, the slight fluctuation of As contents found in the AC profile may result from lithogenic inputs than to cultivation effects, indicating that the influence of agricultural practices on total As content is limited. Nevertheless, artificial management in the field can be another factor to disturb metal distribution. The plowing process during cultivation may bring metals from deeper soils to the tillage layer (0 ∼20 cm) (Wang et al., 2016). The obvious spatial homogeneity of As contents found in the shallow soil of the AC profile may result from the plowing.

**Figure 4:**
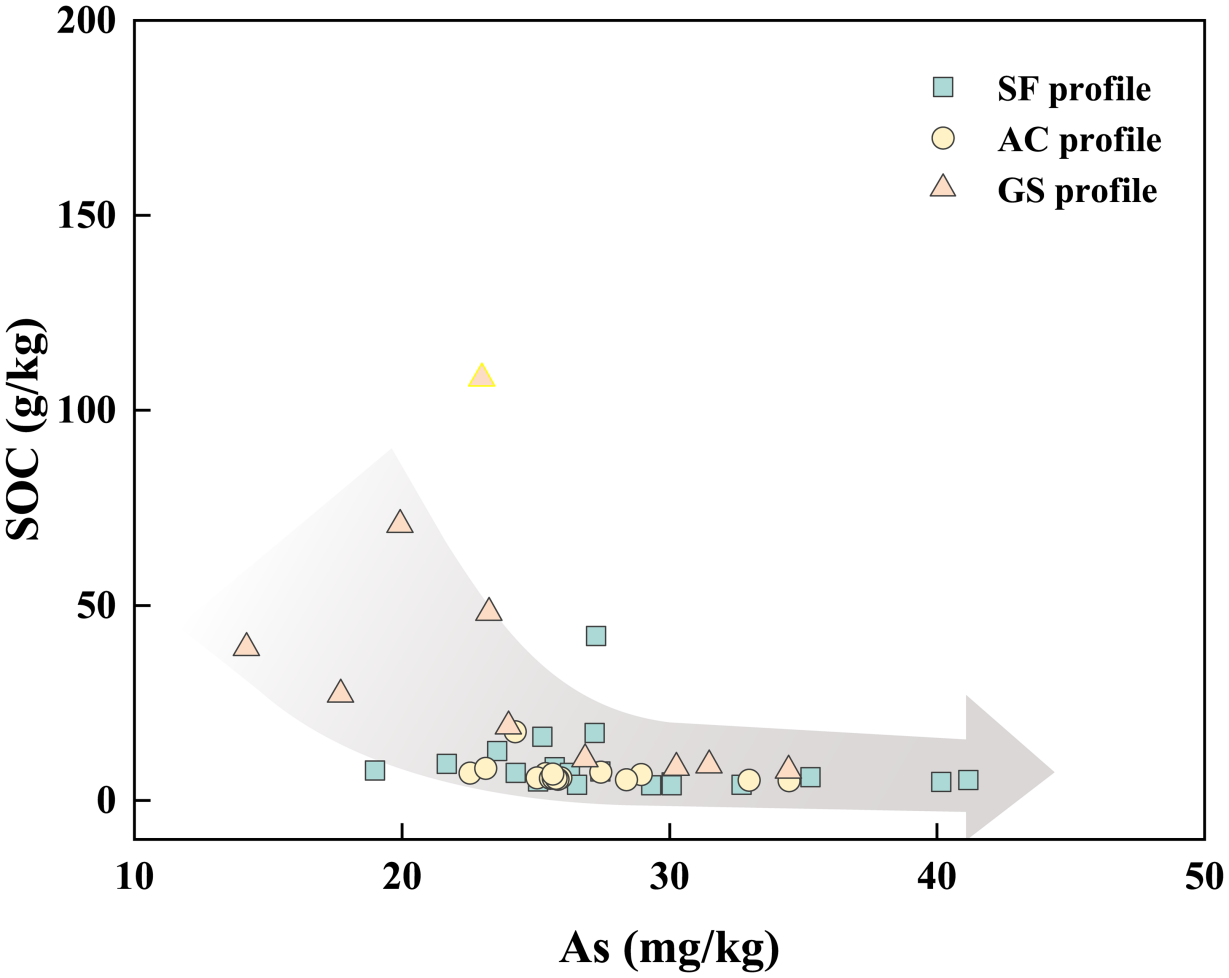
The relationship between the contents of As and SOC in the Yinjiang catchment.

Soil As concentrations exhibit an opposite trend to SOC contents (Fig. 4), indicating that higher organic carbon levels may be associated with enhanced As mobility rather than retention. Compared with the cultivated profile (AC), where tillage tends to homogenize the plow layer and constrain vertical variability, grazing activities in the GS profile appear to broaden the depth distribution of As. Besides inorganic As, organic As possibly may enter to soil environment through the feed assimilation and excreta of goats (Gupta et al., 2018). Organic As-contained additives widely mix into animal feed due to the role of limiting tissue parasitism, improving production rates, and brightening the fur (Gupta et al., 2018; Wang et al., 2010). However, organic As is difficult to absorb by animals and most of them will excreta in the original form (Nookabkaew et al., 2016). As are easily dissolved and migrated in soil with high levels of SOC (Sarkar & Datta, 2004). In addition, SOC may compete with As for the binding sites on the surface of minerals, especially in the organic-rich soils (Bowell, 1994). The SOC contents were at the highest level among the three profiles with an average of 34.8 g/kg in the GS profile. Although organic matter has a large specific surface area and strong adsorption capacity that can theoretically retain substantial amounts of As, microbial decomposition of organic matter under reducing conditions may release previously adsorbed As into the soil solution (Masscheleyn, Delaune & Patrick Jr, 1991; Zhang et al., 2017). Under fluctuating redox conditions, As can be reduced to more mobile As^3+^ in anaerobic zones and re-oxidized and retained in aerobic zones, leading to complex depth distributions (Smith, Jankowski & Sammut, 2003). The relatively reducing environment inferred for the GS profile may therefore enhance As mobility (Han, Zhang & Xu, 2023b), promoting downward migration and accumulation in deeper soil layers. Consistently, As concentrations show a negative correlation with SOC in the GS profile, suggesting that organic matter decomposition facilitates the release of As into the soil solution. After leaching from surface horizons, As may accumulate in deeper layers where adsorption capacity increases due to higher clay contents and greater abundance of Fe/Al oxides.

### Potential hazard of karst soil As on groundwater and residents

**Figure 5:**
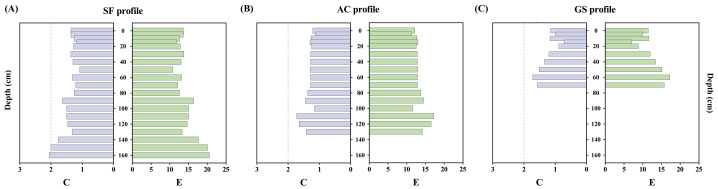
Potential ecological risk (contaminated factor, C; potential ecological risk, E) of SF (A), AC (B), and GS (C) profiles in the study area.

The intake of As has reached 42 µg/day per person in China, which is equivalent to 4 to 4.7 times the sum of As intake in American, Canadian, Australian, and French (Li et al., 2011). It is necessary to make an ecological risk assessment of the soil environment because of the high toxicity coefficient of As, especially in the karst area with high environmental sensitivity. A previous study reported only a mobile fraction of soil As can cause health risks and the migrations are easily affected by soil pH (Pantsar-Kallio & Manninen, 1997). Soil acidification induced by agricultural activities may provide a suitable environment for the diffusion of As in the leaching process. Results showed that most indexes of potential ecological risk (C and E) were lower than the threshold values to threaten the soil environment (C > 2, E > 40) in the study area (Fig. 5). Furthermore, both the values of C and E showed higher levels in deeper soil depth than those in shallow soil (*e.g.*, values of C at 150 cm and 160 cm in SF profile were higher than 2). Compared with the agricultural land, the natural forest land (SF profile) presented higher potential ecological risk (mean value: *C* = 1.4, *E* = 13.6), which indicated that a lower proportion of sand and alkaline soil environment may improve the As accumulation. Although the C and E values of soil As in the study area did not reach the threshold that induced ecological threats, the potential hazard to the soil environment cannot be evaluated in terms of a single element. The superimposed effects of PTEs are strongly adverse to soil ecological balance (Han & Li, 2025; Kang et al., 2020; Qu et al., 2021). A small amount of superposition of As possibly strongly threatens the soil quality in the study area.

Additionally, the enrichment of As with depth is noteworthy in the karst soil profiles and may have implications for subsurface geochemical processes. However, the current study only analyzed solid-phase soils, and no porewater or groundwater samples were collected. Therefore, the potential influence of soil As on groundwater quality cannot be directly confirmed. In karst regions, the presence of fractures, macropores, and preferential flow paths may facilitate vertical migration of solutes, suggesting that the observed depth enrichment could represent a potential source for subsurface transport rather than direct evidence of groundwater contamination. All measured As concentrations in the studied soils are below the Chinese soil pollution risk screening value (GB15618-2018, risk screening value for agricultural land = 25 mg/kg), indicating that the soils do not exceed the regulatory threshold. Nevertheless, the increasing trend of As with depth suggests that redistribution within the soil profile may occur, possibly controlled by redox processes, adsorption–desorption reactions, or mineral associations (Düring, Hoß& Gäth, 2002; Kumarathilaka et al., 2018). High levels of Fe/Mn-oxides in karst soil can easily reduce arsenate to arsenite in porewater, and further be assimilated by plants (Lee, Chiang & Houng, 1996). Frequent fluctuations of groundwater levels in karst environments may enhance the exchange of dissolved components between soil and the subsurface system (Chu et al., 2025; Rudzianskaite & Sukys, 2008). Although the direct measurements of porewater or groundwater were still lack, these processes should be regarded as possible mechanisms. Furthermore, the contrast between anionic As and cationic metals highlights the need for differentiated management strategies. While cationic metals can often be stabilized by increasing pH or organic matter content, the mobility of As is strongly controlled by redox state and Fe-oxide interactions (Kumarathilaka et al., 2018). Therefore, continuous monitoring of soil As dynamics in karst agroecosystems is necessary to better evaluate long-term ecological risks.

**Figure 6:**
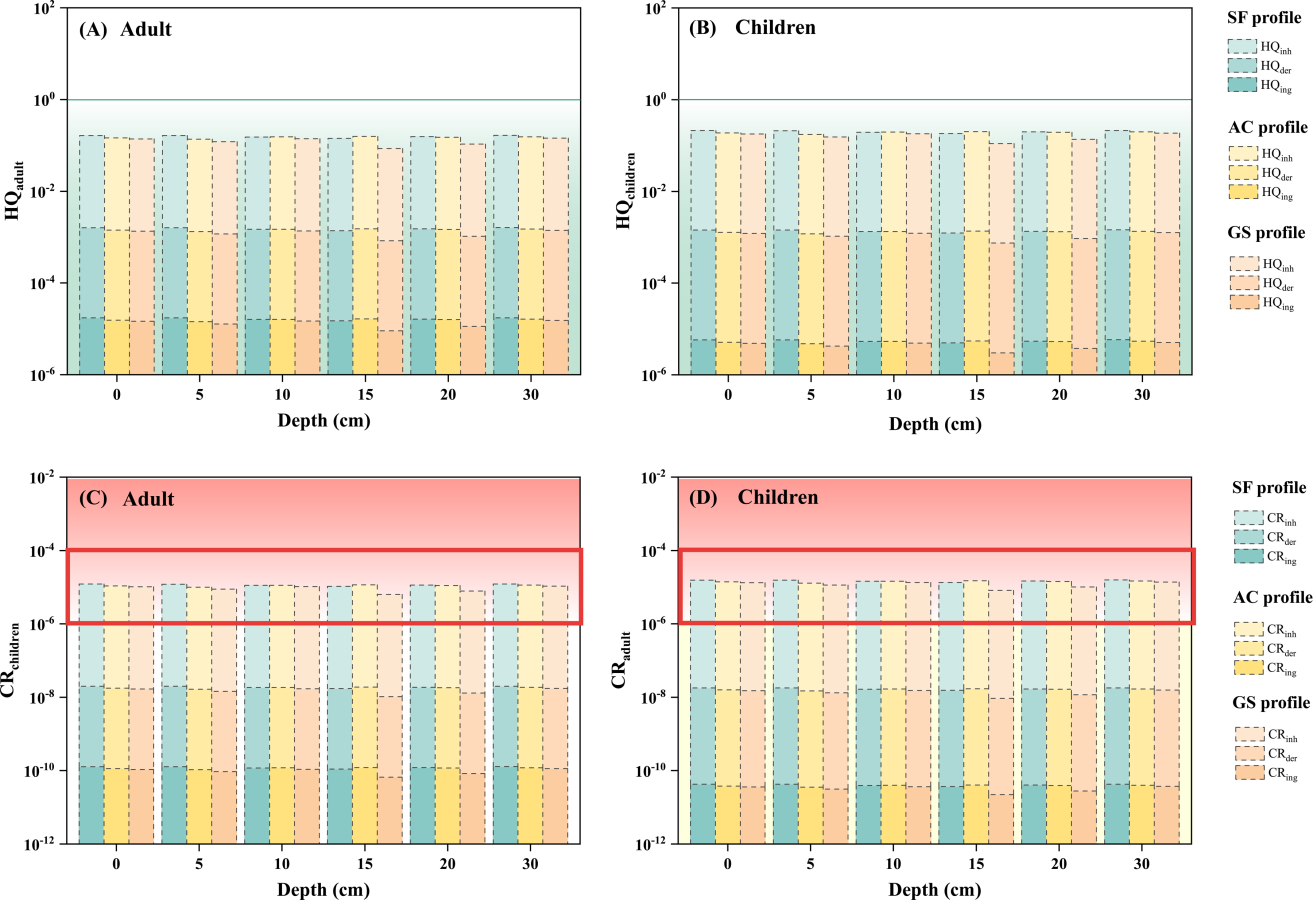
Variation of HQ values for adults (A) and children (B), and CR values for adults (C) and children (D) based on As under diverse land uses.

Surface soils (0 ∼30 cm) under different land uses were selected for human health risk assessment as this is the highest/primary risk of human exposure (Fig. 6). The non-carcinogenic risk was evaluated using the HQ, defined as the ratio between the estimated daily intake and the reference dose for each exposure pathway. The hazard index (HI) represents the sum of HQ values for all exposure routes, and HI < 1 indicates that non-carcinogenic risk is considered acceptable. For adults, the HQ values for inhalation range from 1.28  ×  10^−10^ to 1.73  ×  10^−5^, dermal contact from 1.98  ×  10^−8^ to 1.58  ×  10^−3^, and ingestion from 1.21  ×  10^−5^ to 1.63  ×  10^−1^. For children, the HQ values are consistently lower, with inhalation ranging from 4.27  ×  10^−11^ to 5.78  ×  10^−6^, dermal contact from 1.79  ×  10^−8^ to 1.43  ×  10^−3^, and ingestion from 1.56  ×  10^−5^ to 2.09  ×  10^−1^. In all cases, ingestion was the dominant exposure pathway. The calculated HI values for all soil profiles were lower than 1, indicating that non-carcinogenic health risk from soil As exposure is within the acceptable range for both adults and children. Although HI values for children were generally higher than those for adults within the same soil layer, they still remained below the risk threshold, suggesting increased sensitivity but not an unacceptable risk level. The higher susceptibility of children is consistent with previous studies due to their lower body weight and higher soil intake rate (Chen et al., 2015).

Carcinogenic risk was evaluated using the CR for each exposure pathway, and the total carcinogenic risk (CHI) was calculated as the sum of CR values. According to commonly used criteria, CR values between 10^−6^ and 10^−4^ indicate a tolerable or acceptable risk range. For adults, CR values for inhalation range from 1.11  ×  10^−10^ to 1.28  ×  10^−10^, dermal contact from 1.71  ×  10^−8^ to 1.98  ×  10^−8^, and ingestion from 1.05  ×  10^−5^ to 1.21  ×  10^−5^. Similarly, for children, the CR values are inhalation 3.69  ×  10^−11^ ∼4.27  ×  10^−11^, dermal contact 1.55  ×  10^−8^ ∼ 1.79  ×  10^−8^, and ingestion 1.35  ×  10^−5^ ∼1.56  ×  10^−5^. The calculated CHI values were slightly higher than 10^−6^ but well below 10^−4^ for both groups, indicating the moderate carcinogenic risk rather than the high risk level. Children being more susceptible due to their higher exposure rates and sensitivity. In other words, children may experience greater immediate health risks when exposed to contaminated soil. Acute As poisoning can rapidly induce severe adverse effects on multiple organ systems a within short timeframe (Upadhyay et al., 2019). Although the risk levels are within the acceptable range, the results are based on a single element (As) and only consider soil exposure. Although As is highly toxic, As-compounds do not act as cancer initiators but rather as cancer promoters (Bhattacharya et al., 2010). In summary, attention should be paid to the cumulative ecological risks of As and its combined risks with other carcinogenic compounds. Furthermore, additional pathways, such as groundwater intake or food-chain transfer, were not evaluated in this study. Therefore, continued monitoring is recommended to better assess long-term environmental and human health risks in the Yinjiang catchment.

## Conclusions

The primary source of soil As across all land uses is geogenic, derived from the weathering of As- included carbonate bedrock. As is predominantly retained in soils through adsorption onto Fe/Mn oxides and hydroxides, as well as through co-precipitation with Ca^2+^, contributing to its accumulation particularly in deeper soil layers. Long-term cultivation homogenizes As distribution within the plow layer due to tillage, reducing vertical variability, while historical fertilization shows limited contribution to As accumulation. In contrast, grazing activities introduce organic As *via* animal excreta and promote reducing conditions through increased SOC, enhancing As mobility and facilitating its leaching into deeper soil horizons. This poses a heightened risk of groundwater contamination in the hydrologically connected karst system. Ecological risk assessment indicates that the current As concentrations in soils do not exceed the established screening thresholds when evaluated individually, suggesting a generally low ecological risk. However, the possible interaction with other toxic elements and the high mobility of As under certain geochemical conditions highlight the need for cautious interpretation and continued monitoring, especially in karst environments where soil–water exchange is frequent. Human health risk assessment shows that non-carcinogenic risks for both adults and children remain within acceptable limits across all exposure pathways. Nevertheless, the calculated carcinogenic risk falls within the tolerable range but is relatively higher for children, mainly due to their greater exposure rate relative to body weight and higher sensitivity to environmental contaminants. Therefore, although the current risk level is not considered critical, long-term monitoring of As in soils and related environmental media is recommended to ensure environmental safety and public health protection.

##  Supplemental Information

10.7717/peerj.21371/supp-1 Supplemental Information 1 The detail information of the sampling site and analyzed data

## Additional Information and Declarations

### Data Availability

The following information was supplied regarding data availability: The raw measurements are available in the Supplemental File.
